# Separate block-based parameter estimation method for Hammerstein systems

**DOI:** 10.1098/rsos.172194

**Published:** 2018-06-27

**Authors:** Shuo Zhang, Dongqing Wang, Feng Liu

**Affiliations:** 1 College of Automation and Electrical Engineering, Qingdao University, Qingdao, 266071, People's Republic of China; 2 Collaborative Innovation Center for Eco-Textiles of Shandong Province, Qingdao, 266071, People's Republic of China; 3 Department of Industrial Engineering, University of Texas at Arlington, TX 76019, USA

**Keywords:** parameter estimation, Hammerstein systems, separate block, least squares

## Abstract

Different from the output–input representation-based identification methods of two-block Hammerstein systems, this paper concerns a separate block-based parameter estimation method for each block of a two-block Hammerstein CARMA system, without combining the parameters of two parts together. The idea is to consider each block as a subsystem and to estimate the parameters of the nonlinear block and the linear block separately (interactively), by using two least-squares algorithms in one recursive step. The internal variable between the two blocks (the output of the nonlinear block, and also the input of the linear block) is replaced by different estimates: when estimating the parameters of the nonlinear part, the internal variable between the two blocks is computed by the linear function; when estimating the parameters of the linear part, the internal variable is computed by the nonlinear function. The proposed parameter estimation method possesses property of the higher computational efficiency compared with the previous over-parametrization method in which many redundant parameters need to be computed. The simulation results show the effectiveness of the proposed algorithm.

## 1. Introduction

Nonlinear systems are widespread in practical fields [1–5] and theory study [6–10]. Among various nonlinear models, block-oriented nonlinear structures are frequently used due to their flexibility combining nonlinear elements with linear elements, and flexible expressions of the nonlinear parts and/or linear parts [11–14].

For two-block Hammerstein systems with a nonlinear part plus a linear part, almost all the works reported in the literature aim to get the output–input representation of the systems, and perform a parameter identification based on the output–input expression. The two blocks of Hammerstein systems are always coupled together and can be cast into various input–output models, such as over-parametrization models, bilinear models, linear-in-parameter models. Different models induce different identification methods. From the output–input representation, we can estimate the parameters of the nonlinear block and the linear block. The identification methods include: the over-parametrization-based method [15–18], the iterative method [19–23], the blind identification method [24–26], the key term separation method [27–29], the hierarchical identification method [30–33] and the maximum likelihood method [34–37], etc.

**Figure 1.**
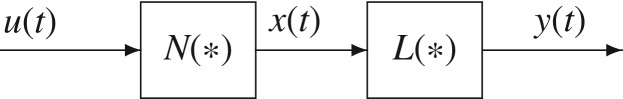
The Hammerstein CARMA system.

Hammerstein systems consist of a nonlinear static block followed by a linear dynamic block, see figure 1.

Previous identification methods are based on an output–input representation of Hammerstein systems.

y(t)=L[N(u(t))].

The investigated method in this paper is based on two separate block representations of Hammerstein systems.

The model of the nonlinear block,

Model 1: x(t)=N[u(t)],

and the model of the linear block,

Model 2: y(t)=L[x(t)].

We perform two least-squares algorithms on these two models, respectively. When identifying Model 1, the internal variable *x*(*t*) is replaced with its estimate, computed from the linear block (Model 2); when identifying Model 2, the internal variable *x*(*t*) is replaced with its estimate, computed from the nonlinear block (Model 1).

The rest of the paper is organized as follows. Section 2 shows the separate block-based least-squares identification algorithms for two blocks of the Hammerstein system. Section 3 provides a numerical example for the proposed algorithms. Finally, the concluding remarks are provided in §4.

## 2. The separate block-based least-squares identification method

Let us introduce some notation. ‘***A*** = :***B***’ stands for ‘***A*** is defined as ***B***’; the symbol ***I*** (***I*_*n*_**) stands for an identity matrix of appropriate size (*n* × *n*); *z* represents a unit forward shift operator: *z*^−1^*x*(*t*) = *x*(*t* − 1); $\hat{z}(t)$ stands for the estimate of *z* at time *t*; the superscript T denotes the matrix/vector transpose; tr[***M***] represents the trace of a square matrix ***M***.

**Figure 2.**
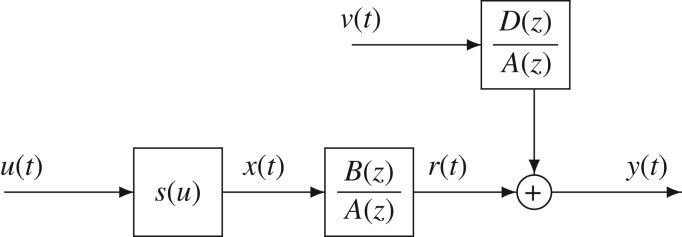
The Hammerstein CARMA system.

The input nonlinear and output linear functions of a Hammerstein CARMA system in figure 2 are expressed as

2.1 $$x(t)=s[u(t)]=\sum_{k=1}^{n_{c}}c_{k}s_{k}[u(t)],$$

and

2.2 A(z)y(t)=B(z)x(t)+D(z)v(t),

where *u*(*t*) and *y*(*t*) are the system input and output, *x*(*t*) is an internal variable, *v*(*t*) is stochastic white noise with zero mean; the input nonlinearity *s* is modelled as a linear combination of basis functions *s*_*k*_, *n*_*c*_ is the number of the basis functions; the linear block is a CARMA model, *A*(*z*), *B*(*z*) and *D*(*z*) are polynomials in the unit backward shift operator *z*^−1^ (*z*^−1^*y*(*t*) = *y*(*t* − 1)), and defined by

$$\begin{array}{rl} A(z) & :=1+a_{1}z^{-1}+a_{2}z^{-2}+\cdots +a_{n_{a}}z^{-n_{a}},\\ B(z) & :=1+b_{1}z^{-1}+b_{2}z^{-2}+\cdots +b_{n_{b}}z^{-n_{b}},\\ D(z) & :=1+d_{1}z^{-1}+d_{2}z^{-2}+\cdots +d_{n_{d}}z^{-n_{d}}. \end{array}$$

Assume the orders *n*_*a*_, *n*_*b*_, *n*_*d*_ are known and *y*(*t*) = 0, *u*(*t*) = 0 and *v*(*t*) = 0 for *t* ≤ 0.

### 2.1. The model of the linear block

Let *y*_1_(*t*) = *y*(*t*) − *x*(*t*), then from the *y* − *x* linear relationship in (2.2), we get

$$\begin{array}{rl} y_{1}(t) & =b_{1}x(t-1)+b_{2}x(t-2)+\cdots +b_{n_{b}}x(t-n_{b})-a_{1}y(t-1)-a_{2}y(t-2)-\cdots -a_{n_{a}}y(t-n_{a})\\ & \;+d_{1}v(t-1)+d_{2}v(t-2)+\cdots +d_{n_{d}}v(t-n_{d})+v(t)\\ & =\varphi_{h}^{T}(t)\theta +v(t), \end{array}$$

where

$$\begin{array}{l} \varphi_{h}(t):=[-y(t-1),\ldots ,-y(t-n_{a}),x(t-1),\ldots ,x(t-n_{b}),\\ \;v(t-1),\ldots ,v(t-n_{d}){]}^{\text{T}}\in {\mathbb{R}}^{n_{a}+n_{b}+n_{d}},\\ \;\theta :={\left[a_{1},a_{2},\ldots ,a_{n_{a}},b_{1},\ldots ,b_{n_{b}},d_{1},d_{2},\ldots ,d_{n_{d}}\right]}^{T}\in R^{n_{a}+n_{b}+n_{d}}. \end{array}$$

Define a least-squares quadratic criterion function

2.3 $$J_{1}(\theta )=\frac{1}{2}\sum_{i=1}^{t}{\left[y_{1}(i)-\varphi_{h}^{T}(i)\theta \right]}^{2}.$$

The internal variable *x*(*t*) and the noise term *v*(*t*) in the information vector ***φ***_*h*_(*t*) are unknown, the solution is to replace them with their estimates, the estimate of *x*(*t*) is computed from the nonlinear part, by replacing *c*_*i*_ with its estimate ${\hat{c}}_{i}(t)$,

$${\hat{x}}_{N}(t)={\hat{c}}_{1}(t)s_{1}[u(t)]+{\hat{c}}_{2}(t)s_{2}[u(t)]+\cdots +{\hat{c}}_{n_{c}}(t)s_{n_{c}}[u(t)],$$

the estimate of *v*(*t*) is computed by

$$\hat{v}(t)=y(t)-{\hat{x}}_{N}(t)-\varphi_{h}^{T}(t)\hat{\theta }(t),$$

then the estimate of ***φ***_*h*_(*t*) is written as

2.4 $${\hat{\varphi }}_{h}(t)={\left[-y(t-1),\ldots ,-y(t-n_{a}),{\hat{x}}_{N}(t-1),\ldots ,{\hat{x}}_{N}(t-n_{b}),\hat{v}(t-1),\ldots ,\hat{v}(t-n_{d})\right]}^{T}.$$

### 2.2. The model of the nonlinear block

From the *x* − *u* nonlinear relationship in (2.1), we get

2.5 $$\begin{array}{rl} x(t) & =c_{1}s_{1}[u(t)]+c_{2}s_{2}[u(t)]+\cdots +c_{n_{c}}s_{n_{c}}[u(t)]\\ & =\varphi_{s}^{T}(t)c. \end{array}$$

where

$$\begin{array}{rl} \varphi_{s}(t) & :={\left[s_{1}[u(t)],s_{2}[u(t)],\ldots ,s_{n_{c}}[u(t)]\right]}^{T}\in R^{n_{c}},\\ c & :={\left[c_{1},c_{2},\ldots ,c_{n_{c}}\right]}^{T}\in R^{n_{c}}. \end{array}$$

Define a least-squares quadratic criterion function

2.6 $$J_{2}(c)=\frac{1}{2}\sum_{i=1}^{t}{\left[x(i)-\varphi_{s}^{T}(i)c\right]}^{2}.$$

*x*(*t*) as an output of the nonlinear block, can be derived from the function (2.2) of the linear block,

2.7 $$x(t)=\frac{A(z)}{B(z)}y(t)-\frac{D(z)}{B(z)}v(t).$$

Let ${\hat{a}}_{i}(t)$, ${\hat{b}}_{i}(t)$ and ${\hat{d}}_{i}(t)$ be the estimates of *a*_*i*_, *b*_*i*_ and *d*_*i*_ at time *t*, the estimates of *A*(*z*), *B*(*z*) and *D*(*z*) at time *t* are

$$\begin{array}{rl} \hat{A}(t,z) & =1+{\hat{a}}_{1}(t)z^{-1}+{\hat{a}}_{2}(t)z^{-2}+\cdots +{\hat{a}}_{n_{a}}(t)z^{-n_{a}},\\ \hat{B}(t,z) & =1+{\hat{b}}_{1}(t)z^{-1}+{\hat{b}}_{2}(t)z^{-2}+\cdots +{\hat{b}}_{n_{b}}(t)z^{-n_{b}},\\ \hat{D}(t,z) & =1+{\hat{d}}_{1}(t)z^{-1}+{\hat{d}}_{2}(t)z^{-2}+\cdots +{\hat{d}}_{n_{d}}(t)z^{-n_{d}}. \end{array}$$

replacing *A*(*z*), *B*(*z*) and *D*(*z*) with their estimates $\hat{A}(t,z)$, $\hat{B}(t,z)$ and $\hat{D}(t,z)$, and *v*(*t*) with $\hat{v}(t-1)$ in (2.7); then from the linear block, the estimate of *x*(*t*) can be written as,

2.8 $${\hat{x}}_{L}(t)=\frac{\hat{A}(t,z)}{\hat{B}(t,z)}y(t)-\frac{\hat{D}(t,z)}{\hat{B}(t,z)}\hat{v}(t-1).$$

### 2.3. The separate block-based least-squares method for two blocks

By using a standard least-squares method, minimizing the cost functions *J*_1_(***θ***) with respect to ***θ*** and replacing *y*_1_(*i*) and ***φ***_*h*_(*i*) with their estimates ${\hat{y}}_{1}(i)$ and ${\hat{\varphi }}_{h}(i)$ in *J*_1_(***θ***), we can obtain the separate block-based least-squares algorithm for the linear block of the Hammerstein CARMA system as follows:

2.9 $$\begin{array}{rl} \hat{\theta }(t) & =\hat{\theta }(t-1)+L_{1}(t)[{\hat{y}}_{1}(t)-\varphi_{h}^{T}(t)\hat{\theta }(t-1)], \end{array}$$

2.10 $$\begin{array}{rl} L_{1}(t) & =P_{1}(t-1)\varphi_{h}(t){\left[1+\varphi_{h}^{T}(t)P_{1}(t-1)\varphi_{h}(t)\right]}^{-1}, \end{array}$$

2.11 $$\begin{array}{rl} P_{1}(t) & =[I-L_{1}(t)\varphi_{h}^{T}(t)]P_{1}(t-1),\;P_{1}(0)=p_{0}I, \end{array}$$

2.12 $$\begin{array}{rl} {\hat{\varphi }}_{h}(t) & ={\left[-y(t-1),\ldots ,-y(t-n_{a}),{\hat{x}}_{N}(t-1),\ldots ,{\hat{x}}_{N}(t-n_{b}),\hat{v}(t-1),\ldots ,\hat{v}(t-n_{d})\right]}^{T}, \end{array}$$

2.13 $$\begin{array}{rl} {\hat{y}}_{1}(t) & =y(t)-{\hat{x}}_{N}(t-1), \end{array}$$

2.14 $$\begin{array}{rl} {\hat{x}}_{N}(t) & ={\hat{\varphi }}_{s}^{T}(t)\hat{c}(t), \end{array}$$

2.15 $$\begin{array}{rl} \hat{v}(t) & =y(t)-{\hat{x}}_{N}(t)-\varphi_{h}^{T}(t)\hat{\theta }(t), \end{array}$$

2.16 $$\begin{array}{rl} \;\text{and}\;\hat{\theta }(t) & ={\left[{\hat{a}}_{1}(t),\ldots ,{\hat{a}}_{n_{a}}(t),{\hat{b}}_{1}(t),\ldots ,{\hat{b}}_{n_{b}}(t),{\hat{d}}_{1}(t),\ldots ,{\hat{d}}_{n_{d}}(t)\right]}^{T}. \end{array}$$

Minimizing the cost function *J*_2_(***c***) with respect to ***c*** and replacing *x*(*i*) with its estimate ${\hat{x}}_{L}(i)$ in *J*_2_(***c***), we can obtain the separate block-based least-squares algorithm for the nonlinear block as follows:

2.17 $$\begin{array}{rl} \hat{c}(t) & =\hat{c}(t-1)+L_{2}(t)[{\hat{x}}_{L}(t)-\varphi_{s}^{T}(t)\hat{c}(t-1)], \end{array}$$

2.18 $$\begin{array}{rl} L_{2}(t) & =P_{2}(t-1)\varphi_{s}(t){\left[1+\varphi_{s}^{T}(t)P_{2}(t-1)\varphi_{s}(t)\right]}^{-1}, \end{array}$$

2.19 $$\begin{array}{rl} P_{2}(t) & =[I-L_{2}(t)\varphi_{s}^{T}(t)]P_{2}(t-1),\;P_{2}(0)=p_{0}I, \end{array}$$

2.20 $$\begin{array}{rl} \varphi_{s}(t) & ={\left[s_{1}[u(t)],s_{2}[u(t)],\ldots ,s_{n_{c}}[u(t)]\right]}^{T}, \end{array}$$

2.21 $$\begin{array}{rl} {\hat{x}}_{L}(t) & =\frac{\hat{A}(t,z)}{\hat{B}(t,z)}y(t)-\frac{\hat{D}(t,z)}{\hat{B}(t,z)}\hat{v}(t-1), \end{array}$$

2.22 $$\begin{array}{rl} \;\text{and}\;\hat{c}(t) & ={\left[{\hat{c}}_{1}(t),{\hat{c}}_{2}(t),\ldots ,{\hat{c}}_{n_{c}}(t)\right]}^{T}. \end{array}$$

The computation process of the separate block-based least-squares algorithm is summarized as follows:

(1) To initialize, let *t* = 1 and $\hat{\theta }(0)=1_{n_{a}+n_{b}+n_{d}}/p_{0}$, $\hat{c}(0)=1_{n_{c}}/p_{0}$, ***P***_1_(0) = *p*_0_***I***, ***P***_2_(0) = *p*_0_***I***, $\hat{v}(t)=0$, $\hat{x}(t)=0$, *u*(*t*) = 0, *y*(*t*) = 0, for *t* ≤ 0, *p*_0_ = 10^6^.
(2) Collect the input–output data ***u***(*t*) and *y*(*t*), and form ${\hat{\varphi }}_{h}(t)$ and ***φ***_*s*_(*t*) using (2.12) and (2.20), respectively.
(3) Compute ${\hat{y}}_{1}(t)$ using (2.13), ***L***_1_(*t*) using (2.10) and ***P***_1_(*t*) using (2.11), ***L***_2_(*t*) using (2.18) and ***P***_2_(*t*) using (2.19).
(4) Update the parameter estimate $\hat{\theta }(t)$ using (2.9).
(5) Compute ${\hat{x}}_{L}(t)$ using (2.21).
(6) Update the parameter estimate $\hat{c}(t)$ using (2.17).
(7) Compute ${\hat{x}}_{N}(t)$ and $\hat{v}(t)$ using (2.14) and (2.15).

**Figure 3.**
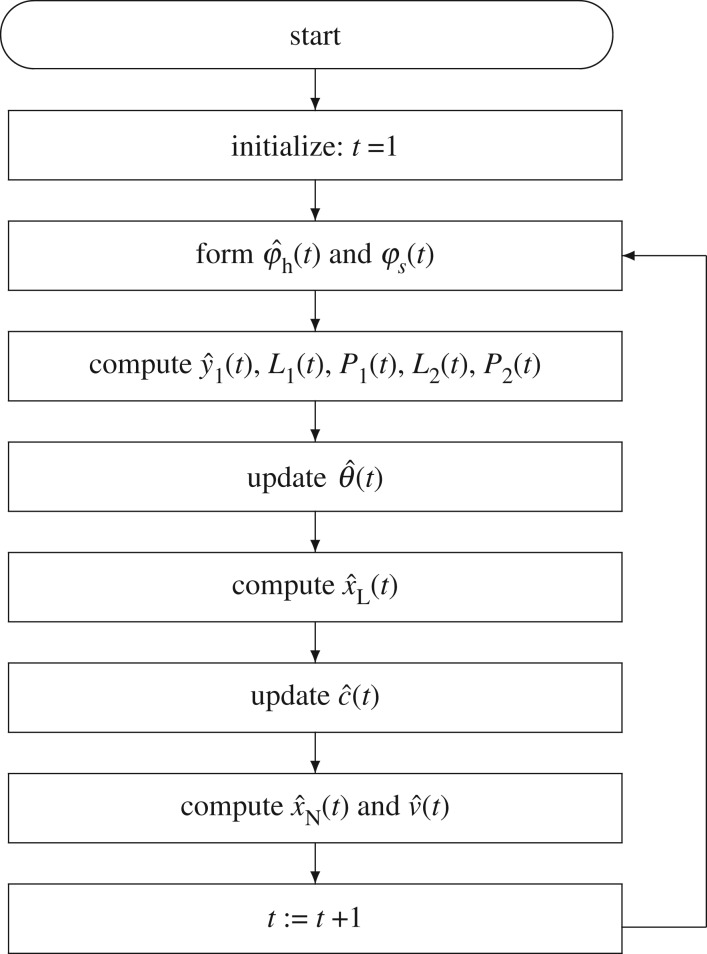
The flowchart of computing the estimates $\hat{\theta }(t)$ and $\hat{c}(t)$.

The flowchart of computing the parameter estimates $\hat{\theta }(t)$ and $\hat{c}(t)$ using the separate block-based least-squares algorithm in (2.9)–(2.22) is shown in figure 3.

## 3. Results

Consider the following Hammerstein CARMA system

$$\begin{array}{rl} A(z)y(t) & =B(z)x(t)+D(z)v(t),\\ x(t) & =c_{1}u(t)+c_{2}u^{2}(t)+c_{3}u^{3}(t)\\ & =0.65u(t)+0.90u^{2}(t)+1.25u^{3}(t),\\ A(z) & =1+a_{1}z^{-1}+a_{2}z^{-2}=1+1.55z^{-1}+0.90z^{-2},\\ B(z) & =1+b_{1}z^{-1}+b_{2}z^{-2}=1.00-0.50z^{-1}+0.75z^{-2},\\ D(z) & =1+d_{1}z^{-1}=1+0.30z^{-1},\\ a & ={\left[a_{1},a_{2}\right]}^{T}={\left[1.55,0.90\right]}^{T},\\ b & ={\left[b_{1},b_{2}\right]}^{T}={\left[-0.50,0.75\right]}^{T},\\ d & =d_{1}=0.30,\\ c & ={\left[c_{1},c_{2},c_{3}\right]}^{T}={\left[0.65,0.90,1.25\right]}^{T},\\ \theta & ={\left[a^{T},b^{T},d^{T}\right]}^{T}. \end{array}$$

In simulation, the input {*u*(*t*)} is taken as an uncorrelated persistently excited signal vector sequence with zero mean and unit variance, and {*v*(*t*)} is taken as a white noise sequence with zero mean and variances *σ*^2^ = 1.00^2^ and *σ*^2^ = 3.00^2^. Applying the proposed separate block-based least-squares algorithm to estimate the parameters of this system, the parameter estimates and their errors are shown in table 1. The estimation error of the parameters is

$$\delta :=\sqrt{\frac{∥\hat{a}(t)-a{∥}^{2}+∥\hat{b}(t)-b{∥}^{2}+∥\hat{c}(t)-c{∥}^{2}+∥\hat{d}(t)-d{∥}^{2}}{∥a{∥}^{2}+|b{∥}^{2}+|c{∥}^{2}+∥d{∥}^{2}}}\times 100\%.$$

**Table 1.** The parameter estimates and errors.

*σ*^2^	*t*	${\hat{a}}_{1}(t)$	${\hat{a}}_{2}(t)$	${\hat{b}}_{1}(t)$	${\hat{b}}_{2}(t)$	${\hat{c}}_{1}(t)$	${\hat{c}}_{2}(t)$	${\hat{c}}_{3}(t)$	${\hat{d}}_{1}(t)$	*δ* (%)
0.10^2^	100	1.55751	0.90665	−0.52257	0.69811	0.66850	0.91160	1.24310	0.35475	3.14282
	200	1.55541	0.90247	−0.52755	0.73469	0.66850	0.91160	1.24310	0.25586	2.25102
	500	1.54906	0.89975	−0.51690	0.75337	0.66850	0.91160	1.24310	0.30769	1.12955
	1000	1.55041	0.90126	−0.51247	0.74755	0.66850	0.91160	1.24310	0.34413	1.95302
	2000	1.55112	0.90180	−0.50225	0.73870	0.66850	0.91160	1.24310	0.31593	1.15119
	3000	1.55073	0.90212	−0.50502	0.74539	0.66850	0.91160	1.24310	0.28467	1.08345
0.15^2^	100	1.54905	0.90332	−0.61210	0.66692	0.66850	0.91160	1.24310	0.34304	5.62443
	200	1.55438	0.89568	−0.60168	0.73386	0.66850	0.91160	1.24310	0.24995	4.44735
	500	1.54738	0.89744	−0.55301	0.76738	0.66850	0.91160	1.24310	0.30463	2.30518
	1000	1.55151	0.90371	−0.53975	0.74896	0.66850	0.91160	1.24310	0.34611	2.47946
	2000	1.55292	0.90477	−0.50917	0.72224	0.66850	0.91160	1.24310	0.31749	1.57603
	3000	1.55108	0.90512	−0.51753	0.74260	0.66850	0.91160	1.24310	0.28457	1.29130
true values		1.55000	0.90000	−0.50000	0.75000	0.65000	0.90000	1.25000	0.30000	

From table 1 we can get: (1) the parameter estimation errors become (generally) smaller and smaller with the recursion *t* increasing; (2) the parameter estimates converge to their true values as the noise variance becomes small.

## 4. Conclusion

This paper concerns a separate block-based parameter identification method for each block of the Hammerstein CARMA system, without forming the whole output–input representation of the system. The idea is to consider each block separately as a subsystem in the Hammerstein system and to estimate the parameters of the nonlinear block and the parameters of the linear block separately (interactively), by using two least-squares algorithm in one recursive step. The internal variable between the two blocks is replaced by different estimates: for estimating the parameters of the nonlinear part, the internal variable is computed by the linear function; for estimating the parameters of the linear part, the internal variable is computed by the nonlinear function.

The proposed simple parameter estimation method possesses property of the higher computational efficiency compared with the previous over-parametrization method in which many redundant parameters need to be computed, and can be extended to systems with more blocks [38] and networked dynamic systems [39,40], etc.

## Data accessibility

The data from this article are available from the Dryad Digital Repository: (http://dx.doi.org/10.5061/dryad.k49ht50) [41].
